# 3D Printed Polyvinyl Alcohol Tablets with Multiple Release Profiles

**DOI:** 10.1038/s41598-019-48921-8

**Published:** 2019-08-28

**Authors:** Xiaowen Xu, Jingzhou Zhao, Maonan Wang, Liang Wang, Junliang Yang

**Affiliations:** 1 School of Physics and Electronics, Central South University, Changsha, Hunan 410083 China; 2 Department of Bioinformatics, School of Medical Informatics and Engineering, Xuzhou Medical University, Xuzhou, Jiangsu 221000 China; 3 Jiangsu Key Laboratory of New Drug Research and Clinical Pharmacy, School of Pharmacy, Xuzhou Medical University, Xuzhou, Jiangsu 221000 China

**Keywords:** Drug delivery, Pharmaceutics, Engineering

## Abstract

The aim of this study was to explore the feasibility of using different 3D printed internal geometries as tablet formulations to obtain controlled release profiles. In order to obtain controllable release profiles, three types of tablet models (Cylinder, Horn and Reversed Horn) with controlled structures were designed. The cylinder model shows a constant release profile and can keep the drug concentration within a certain range. The horn model exhibits an increasing release profile, which is suitable for the patients who have the drug resistance in the course of medication. The reversed horn model has a decreasing release profile that would be applied to hypertension cure. Furthermore, three types of tablets were fabricated successfully by a fused deposition modeling three-dimensional (3D) printer and injected with paracetamol (APAP) -containing gels. The results of *in vitro* drug release demonstrate that tablets with three kinds of structures can produce constant, gradually increasing, and gradually decreasing release profiles, respectively. The release attributes can be controlled by using different 3D printed geometries as tablet formulations. More importantly, there are no residues after dissolution. The method of preparing customized tablets with distinguished release profiles presented in this study has the promising potential in the fabrication of patient-tailored medicines.

## Introduction

Three-dimensional (3D) printing or additive manufacturing is a rapid prototyping technology that prints 3D objects by layer-by-layer deposition approach controlled by computer-aided design software^1^. 3D printing has been used to generate complex structures, which are very challenge to manufacture with traditional techniques. It has been studied in the medical and pharmaceutical applications such as tissue engineering^2–5^, dentistry^6^, and implants^7,8^. Tablets with different structures and materials will have distinguished release profiles^9^. In August 2015, the application of 3D printing in the pharmaceutical industry was approved by US Food and Drug Administration (FDA)^9,10^, indicating that 3D printing could elaborate its advantages in personalized medicine.

Recently many researchers have paid more efforts to employ 3D printing to develop personalized medicines^11–13^ and drug delivery system^14–21^. There are multiple 3D printing technologies utilized in customized medicines, such as Stereolithography (SLA)^22^, Selective Laser Sintering (SLS)^23,24^, Fused Deposition Modelling (FDM)^13,25,26^, Semi-solid extrusion (SSE)^27^ and Powder Based (PB)^28^. 3D printing is used to fabricate oral dosage forms and demonstrates great potentials in the pharmaceutical industry^12,13,29,30^. Currently, researchers fabricated controlled-release drugs with different characteristics by 3D printing^22,30–36^. Tablets with different structures and materials will have distinguished release profiles^9^. Yu *et al*. employed PB 3D printing to develop doughnut-shaped multi-layered tablets with linear release profile^28^. Wang *et al*. used SLA 3D printer to manufacture drug-loaded tablets with modified-release characteristics and revealed that the release profile of the drugs was dependent on the composition of the formulations^22^. Trenfield *et al*. fabricated printlets with three different geometries using a desktop SLS printer and developed a calibration model to predict drug concentration of a different geometry^37,38^. Of these 3DP technologies FDM technology exhibits the excellent promise in fabricating drug products, because FDM printers are low-cost, easy to operate^9^ and able to refine hollow objects^39^. FDM 3D printing has recently attracted increasing interests as one of the most widely used techniques for developing individualized medicines in pharmaceutical applications^13^. Muwaffak *et al*. used 3D scanning to construct models of a nose and ear and printed a customized wound dressing using antimicrobial metals incorporated into polycaprolactone (PCL) to produce filaments for 3D printing^40^. These metals with broad-spectrum antimicrobial properties can improve the wound healing process^41,42^. FDM 3D printing is an effective process to fabricate tablets with modified release profiles. FDM printer has been used to produce immediate, sustained, and time-released tablets^43^. It is able to manufacture complex shapes and geometries to obtain different release profiles in personalized medicine^9,44^. Skowyra *et al*. used FDM printer to produce prednisolone sustained-release tablets^13^. Melocchi *et al*. explored the potential of FDM 3D printing to manufacture oral capsular devices for pulsatile release^25^. Chai *et al*. explored the potential of FDM 3D printing to prepare sustained-release drugs, which could be released in the stomach for a long time^45^. Goyanes *et al*. produced tablets with different geometrical shapes by FDM 3D printing and demonstrated that tablet shape could affect drug release profile^44^.

Hydroxypropyl methylcellulose (HPMC), a non-toxic and safe pharmaceutical excipient, has been employed as the forming agents and binding additives in order to obtain sustained release^33,46–49^. Zhang *et al*. explored the relationship between drug release and the geometrical shape of the 3D printed tablets with different structures printed by FDM printing^50^. The desktop 3D printer was used to produce bilayer tablets with a definable release profile through a hydrated HPMC gel layer^12^. Khaled *et al*. reported 3D extrusion printing of a complex multi-compartment tablet^12,51^. Kadry *et al*. employed HPMC and diltiazem to prepare both drug-free and drug-impregnated filaments and printed tablets with different infill densities and patterns^49^. However, the shrinkage of the gel model affected the shape of the printed tablets^13^. The hot melt extrusion (HME) process is indispensable in FDM 3D printing, which allows the thermally softened filaments to be extruded by a nozzle and to be deposited layer-by-layer^52^. Genina *et al*. investigated ethylene vinyl acetate (EVA) as new feedstock material for FDM 3D printing technology to print medical drug delivery devices^53^. Polyvinyl alcohol (PVA) is one of most widely used water-soluble synthetic polymer in various applications^54–59^. It has been utilized as benchmark polymer^60^ and drug carriers^30,34,61,62^ in 3D printed pharmaceuticals because of its good biocompatibility. Pluta *et al*. described the application of polyvinyl alcohol (PVA) in the technology of modern drug form^63^. Goyanes *et al*. used a filament extruder to obtain filaments of PVA containing paracetamol (APAP) and fabricated PVA-based caplets with specific release profiles by FDM 3D printing^64^. Tagami *et al*. produced composite tablets with a drug-loaded PVA component and a PVA or PLA filler component using FDM 3D printer with dual nozzles^65^. The major shortcoming of FDM 3D printing in pharmaceutical applications is the elevated extrusion and printing temperatures required in the printing process, which limits its application in 3D printing drugs^41,49,66,67^. So, it is not suitable to produce thermolabile drugs because the pharmaceutical excipients and active drugs may degrade at high temperature during the extrusion and printing processes^30,51,60^. Many investigators have attempted to address this issue. Kollamaram *et al*. printed ramipril printlets by reducing the FDM printing temperature to 90 °C^48^. Kempin *et al*. printed dual coated tablets by dual-extrusion-printing using three-part printing designs and utilized polycaprolactone (PCL) as the coating with a printing temperature of 58 °C^68^. However, the printing process is complex and PCL is nearly insoluble. Sun *et al*. incorporated insoluble containers and light curing materials into a tablet and obtained the release profile of the drug via controlling the area of the effective component to release in accordance with the scheduled time^36^. However, the challenge of preparing tablets with this method is the insoluble container and the complex manufacturing process. The main reason for limiting FDM 3D printing in pharmaceutical applications is that the 3D printing need to mix the drug to the polymeric filament and heat drug loading filament to a high temperature to extrude through a nozzel and deposit layer by layer.

We recently reported the fabrication of a tablet with a regular tetrahedron cavity printed by a 3D printer^69^. The support structure (shell) and the drug were fabricated separately. PVA filaments were printed as a soluble container and PVA gel containing drugs was injected into the cavity at room temperature. The tablet with a regular tetrahedron cavity can only provide one kind of drug release profile, *i.e*., increasing release profile.

The aim of this study is to explore the use of different 3D printed internal geometries as tablet formulations to obtain multiple controlled-release profiles. PVA scaffolds with different internal architecture were printed by a commercially 3D printer and injected with drug-containing gel. Tablets produced by our method have the advantages of multiple release profiles, high temperature resistance, and no residues after dissolution.

## Results and Discussion

### Establishment of three tablet models

**Figure 1:**
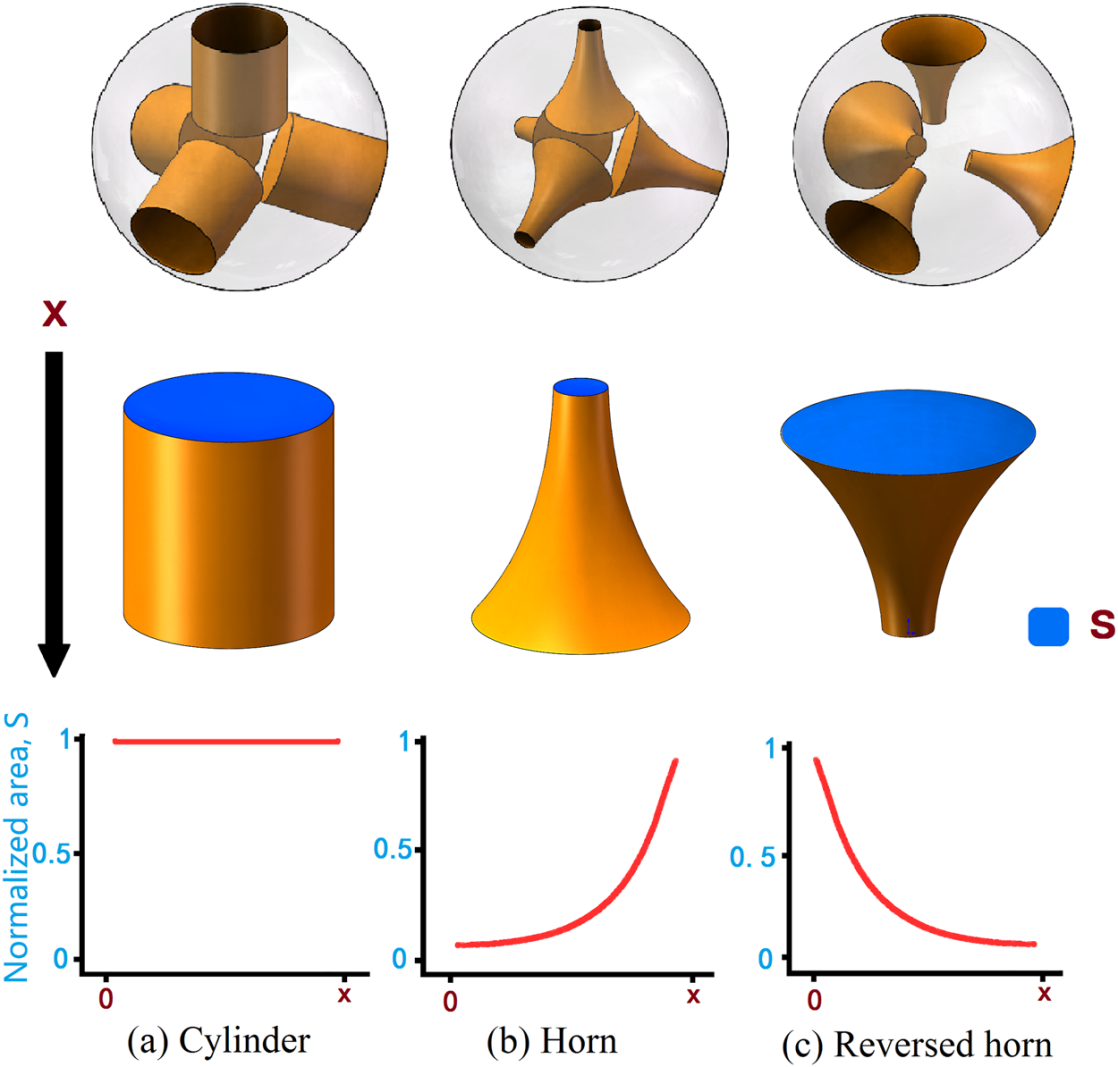
Three kinds of models of tablet kernels with different shapes. (**a**) The first model has four cylinder cavities placed in a sphere (Cylinder model). (**b**) The second model has four horn cavities placed in a sphere (Horn model). (**c**) The third model has four reversed horn cavities placed in a sphere (R-Horn model). The blue part represents the exposed area of the drug kernel. Along with the decrease of height, the surface area (*S*) will change in three kinds of models.

Figure 1 shows three kinds of models of tablet kernels with different shapes, *i.e*., Cylinder, Horn and Reversed Horn (R-Horn). Three types of cavities are embedded in the sphere for achieving different release profiles. In order to use more effective components and also make the drug release more uniformly, four symmetrically distributed cavities are designed and evenly placed in a sphere with a radius of 6 mm. *X* represents the height of cavities. Cavities will be filled with drug gel by injection. Tablets with different models will have distinct release attributes. The surface area *S* of the exposed drug will be constant, gradually increasing and gradually decreasing for Cylinder model, Horn model and R-Horn model, respectively. The inner architecture of the tablet would be gradually exposed with the decrease of the diameter of the tablet (Fig. S1) and the release rate would be different due to the change of surface area of medicine core.

### Preparation of tablets

**Figure 2:**
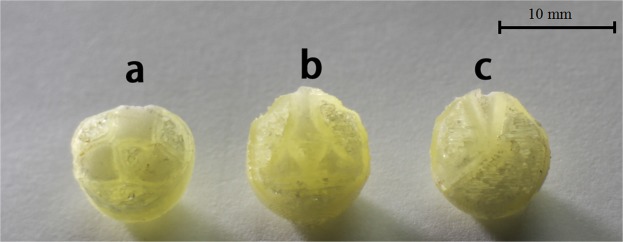
Photos of 3D printed tablets with the three models. (**a**) Cylinder model, (**b**) Horn model, (**c**) R-Horn model.

**Figure 3:**
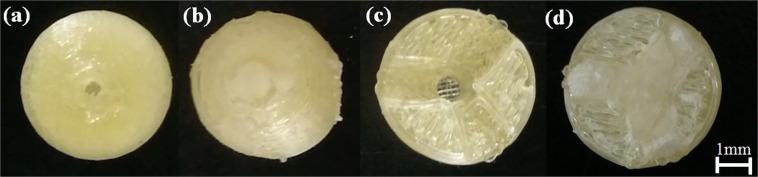
The structure of the tablet with Cylinder model. (**a**) tablet shell with a hole, (**b**) tablet after injection of the drug molecule, (**c**) cross section of tablet shell before injection, (**d**) cross section of tablet shell after injection.

Figure 2 shows the tablets with Cylinder model, Horn model and R-Horn model printed by FDM 3D printing. The pale yellow part is PVA shell, and the milky white part is APAP-containing gel after drying. The weight and diameter of each tablet were measured. *X, S*_top_ and *S*_bottom_ represent the height of the cavity of each model, the surface area of top side and bottom side of each model in Fig. 1. The parameters of three kinds of tablets are listed in Table 1. Figure 3 shows the structure of pore and inner architecture of the tablet with Cylinder model before and after injection of the drug molecule.

**Table 1:** Parameters of the 3D printed tablets with Cylinder model, Horn model and R-Horn model.

Model	Cavity height/mm	S_top_/mm^2^	S_bottom_/mm^2^	Weight/g	Diameter/mm
Cylinder	4.15 ± 0.14	12.57 ± 0.2	12.57 ± 0.2	0.85 ± 0.026	9.98 ± 0.18
Horn	4.48 ± 0.21	0.97 ± 0.02	17.57 ± 0.28	0.92 ± 0.019	10.20 ± 0.15
R-Horn	4.50 ± 0.24	19.27 ± 0.24	0.79 ± 0.01	0.89 ± 0.016	10.13 ± 0.12

**Figure 4:**
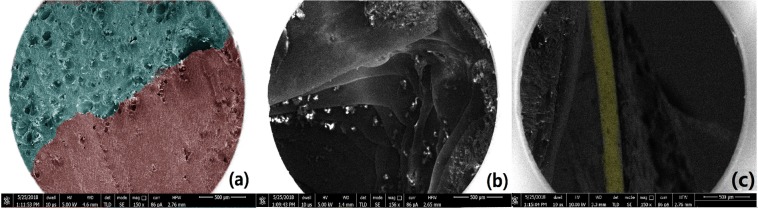
SEM visualization of cross-section of 3D printed PVA tablet with Cylinder model after injection of APAP gel. (**a**) Green part is the APAP gel, red part means PVA. (**b**) the layered structure of the shell, (**c**) The yellow part represents the perimeter of the drug.

 SEM images of cross-section of 3D printed PVA shell with Cylinder model and injected APAP gel are shown in Fig. 4. The fabricated tablet is sufficiently hard and does not collapse during 3D printing. Figure 4a shows that there is no gap between PVA and APAP gel after drying. Figure 4b represents the layered structure of the shell, which is the processing feature of FDM printing. Figure 4c shows a contour line that forms the perimeter of the drug.

### ***In vitro*** drug release study

Tablets with Cylinder, Horn and R-Horn models are fabricated by printing PVA shell with FDM 3D printer and injecting APAP-containing gels into the cavity at room temperature. The phosphate buffer solution (PBS, pH = 6.8, 0.05 M, 900 mL) was used as representing the colonic environment^44^. *In vitro* drug dissolution tests were conducted by putting the tablets in phosphate buffer solution (PBS, pH = 6.8) with stirring (50 rpm) at 37 ± 0.5 °C. The concentration of active ingredient of APAP was measured by an ultraviolet spectrophotometer (UV-2601, Beijing RuiLi Analytical Instrument Co.Ltd, China). Samples were measured every 15 minutes during dissolution. The absorption peaks of APAP and PVA were detected at 243 nm and 264 nm, respectively. By the calculation, the influence of PVA could be removed, and the absorbance of the APAP could be obtained. In this way, the dissolution rate can be obtained by calculating the change of APAP concentration.

**Figure 5:**
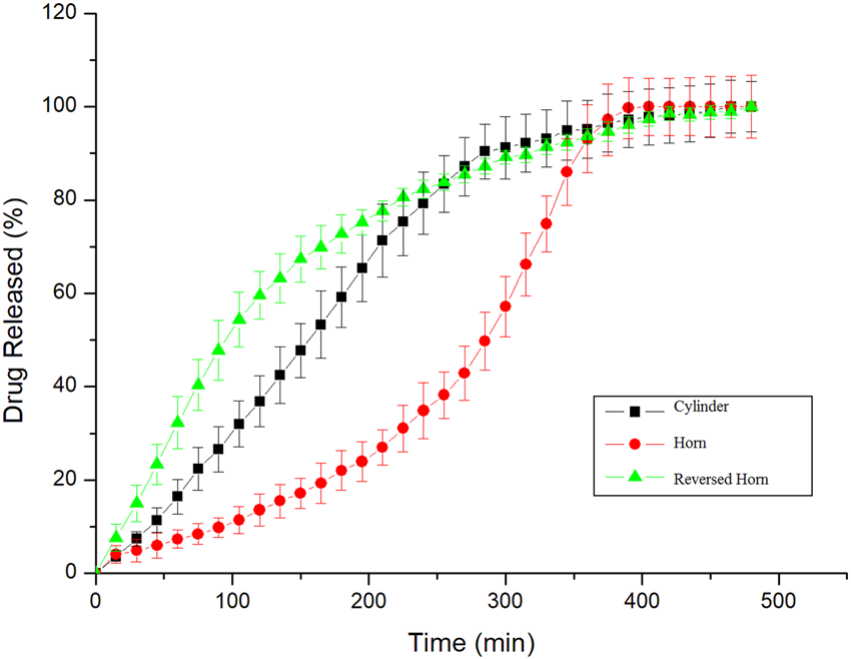
The release profiles of APAP tablets with Cylinder model, Horn model, and R-Horn model.

Tablets gradually melted along with the decrease of tablet diameter during dissolution. The release rate of tablets gradually varied with the change of surface area of tablets. The content of the drug was gradually exposed to PBS, which would facilitate the release rate of tablets. Tablets with three structures were dissolved according to the predetermined patterns of three models. The release was almost completed in ca. 6 hours. The release profiles of 3D printed APAP tablets are shown in Fig. 5. Tablets with three kinds of structures show constant, gradually increasing, and gradually decreasing release rate, respectively. Burst release, which is undesired for controlled-release drugs applications, is not observed from the release profiles of APAP tablets. It may be a result of using higher molecular weight PVA to decrease diffusion pathway^62^. In the previous work, we successfully printed a tablet with increasing release profile^68^. In this study, tablet of each structure has its characteristic release profile during dissolution. The Cylinder model has a constant release profile that can maintain the drug concentration within a certain range. The Horn model has an increasing release profile, which is suitable for patients with drug tolerance in the course of medication and can also be applied to hypertension which often occurs in the morning^70^. The R-Horn model has a decreasing release profile that can be valuable for cases where a large dose of drug is required initially to act against their targets rapidly^36^. The drug release data demonstrate that the internal architectures of tablets have an important effect on drug release rate.

**Figure 6:**
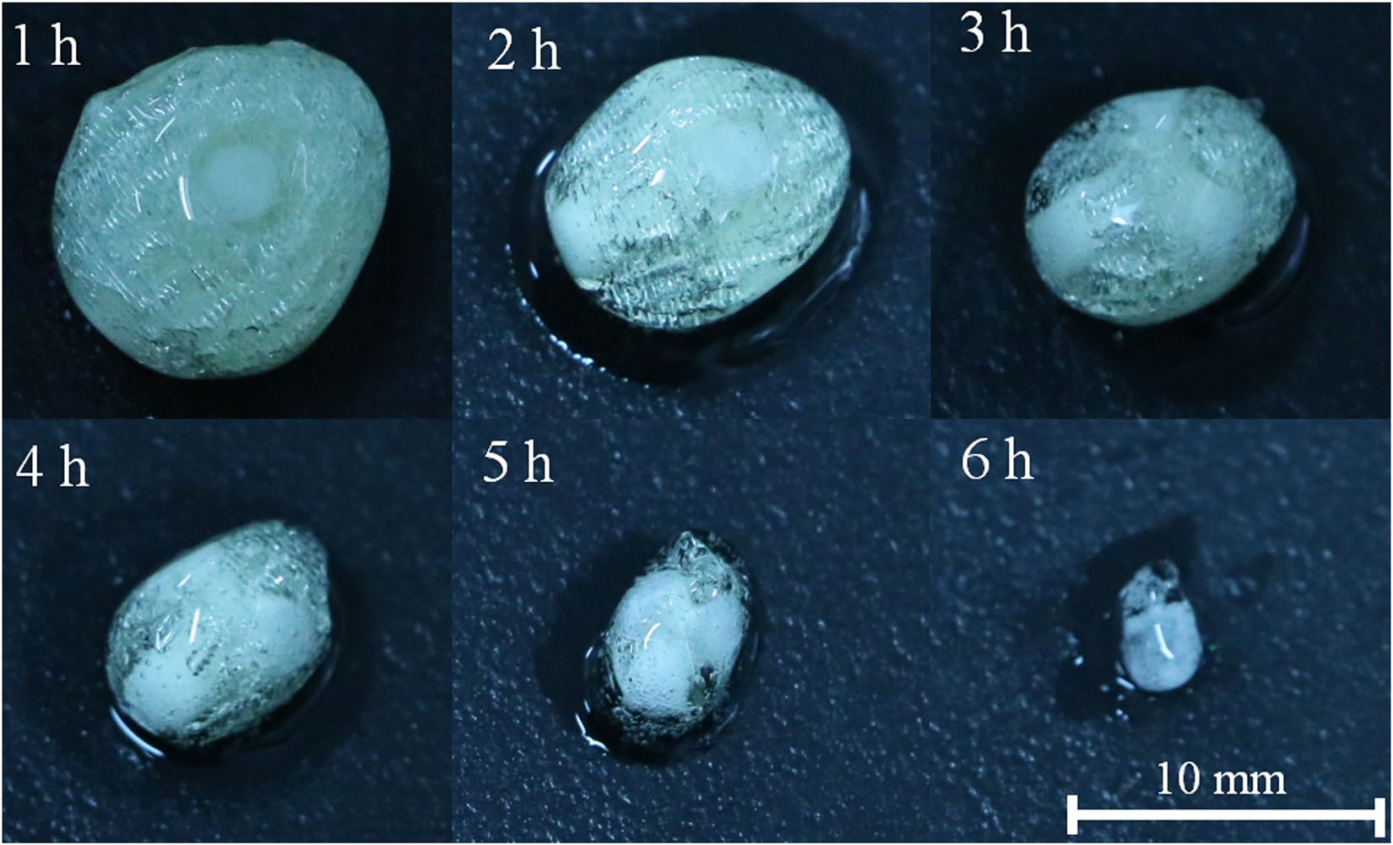
Tablets with Cylinder model during dissolution.

Figure 6 reveals the dissolution of tablets with Cylinder model every 1 hour when dissolved in PBS. Over time, tablets were gradually eroded by PBS and became smaller. Although the drug became softened during dissolution, the inner architecture of scaffolds of the tablet still existed. This is because the dissolution rate of the PVA material is lower than that of the APAP gel. This phenomenon could be explained that drug release was determined by an erosion-mediated mechanism reported by a couple of studies^13,39,44^. In terms of *in vitro* dissolution results, the release rates of the tablets with three models are positively related to the changes of surface area, which is consistent with the results reported by Goyanes^44^. Generally, the release profile of single-component drugs was decreasing because the surface area of active ingredients was uncontrolled. We designed different 3D printed geometries as controlled release system to govern the inner architecture of the tablets to obtain constant, gradually increasing and gradually decreasing release profiles.

This study demonstrates that the potentials of FDM 3D printing technology to fabricate controlled release tablets with different release profiles in accordance with three kinds of models. Meanwhile, PVA can be used for different kinds of medicines, providing a positive effect for personalized medicine. Sun *et al.* used an insoluble container and light curing materials to produce a tablet for getting the drug release profile^36^. We fabricated tablets with distinguished release profiles using PVA filaments as a soluble container filled with PVA gels containing drugs. The tablets in our work were manufactured with simple fabrication process and there is no residue after dissolution because the core and shell of tablets are soluble. In our study the drug-loaded gel was injected into the cavities without high temperature and the active ingredients of the drug were not degraded. Compared to hot melt extrusion by adding APAP into PVA filament to fabricate controlled-release drugs at about 200 °C^32,34^, the way of injecting the drug-containing gel into the cavity at room temperature also avoids high temperature damage to the active ingredients of the tablets, and it is more suitable for the thermo-sensitive tablets. Compared to other 3D printing tablets, our work takes into account more aspects. For example, one of the typical advantages is that there is no insoluble container. Secondly, the manufacturing steps are simple so that it will be more suitable for popularizing on a large scale. Thirdly, our approach is suitable to print thermo-sensitive drugs compared to the method of adding drugs to PVA filament^32^. However, it still has some shortcomings such as short dissolution cycle (less than 7 hours) and lacking of the structure diversity. In the future, it is necessary to develop other gel components to fill the tablet containers so that the dissolution time would be extended.

## Conclusion

We proposed a new method to fabricate customized tablets with distinguished release profiles using 3D printing technology and filling them with drug-containing gel at room temperature, which provides a new way for tailored drugs with simple fabrication process. We designed different 3D printed geometries as tablet formulations for the controlled drug release and explored the effect of inner architecture of scaffolds to obtain constant, gradually increasing or gradually decreasing release rate. The drug release rate depends on internal architectures of tablets. Tablets produced in this work could be dissolved evenly and there was no residue after dissolution. The manufacturing process did not degrade the active ingredients of drugs because drug-containing gels injected into the cavity at room temperature don’t need to be heated high temperature of over 200 °C, which could be applied to other thermolabile tablets. In addition, the simple fabrication process for customized tablets by 3D printing technologies and easily available materials also make this general method suitable for popularization on large scale, which plays an important role in the personalized medicine.

## Materials and Methods

### Preparation of drug gel

Water, APAP (Anta Biotechnology, China) and PVA powder (Yingjia Inc., China) were mixed at a ratio of 6:3:1, stirred evenly for 15 minutes, then put into the vacuum chamber and evacuate until the air pressure is 400 Pa to remove bubbles of the drug gel.

### 3D printing PVA shell

FDM 3D printer (Creator Pro, FlashForge, China) is employed to produce a soluble container of the tablet with PVA filament (MOSHU Inc., China) at the temperature of 180 °C using a nozzle diameter of 0.3 mm at an infill rate of 100% and printing speed of 60 mm/s. It has shown that infill rate can significantly affect the release profile, and the infill rate is set to 100%, excluding the interference of infill contain cavities^63^.

### Injecting gel into PVA shell

**Figure 7:**
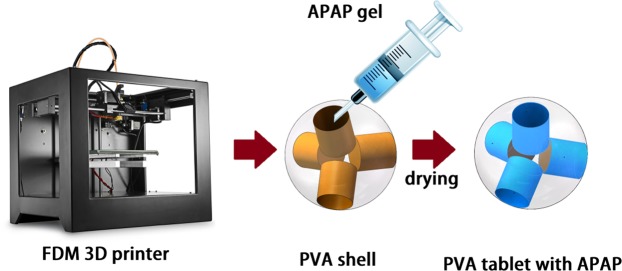
The process of preparing tablets.

The prepared drug gel was injected into the cavity by a 1-mL syringe with a needle (inner diameter of 0.5 mm) through a hole with a diameter of 0.7 mm, which was drilled at the thinnest part of PVA shell (Fig. 7). After injecting, the tablets were dried in a vacuum drying oven at 60 °C for 12 hours.

### Scanning electron microscope (SEM) characterization

In order to investigate whether APAP adheres closely to PVA after injection or not, the cross-section images of the tablet were characterized using SEM (FEI Helios Nanolab 600i SEM, America). The cross section of the tablet was milled by sandpaper.

### Ethical approval

This article does not contain any studies with human participants or animals performed by any of the authors.

## Supplementary information

Supplementary Information

## Data Availability

The datasets generated during and/or analysed during the current study are available from the corresponding author on reasonable request.
