# Author Correction: 3D Printed Polyvinyl Alcohol Tablets with Multiple Release Profiles

**DOI:** 10.1038/s41598-020-59303-w

**Published:** 2020-02-06

**Authors:** Xiaowen Xu, Jingzhou Zhao, Maonan Wang, Liang Wang, Junliang Yang

**Affiliations:** 1 School of Physics and Electronics, Central South University, Changsha, Hunan 410083 China; 2 Department of Bioinformatics, School of Medical Informatics and Engineering, Xuzhou Medical University, Xuzhou, Jiangsu 221000 China; 3 Jiangsu Key Laboratory of New Drug Research and Clinical Pharmacy, School of Pharmacy, Xuzhou Medical University, Xuzhou, Jiangsu 221000 China

Correction to: *Scientific Reports* 10.1038/s41598-019-48921-8, published online 28 August 2019

This Article contains errors in References 1, 20, 45, 56 and 57 which were incorrectly given as:

Xiong, Y. J. *et al*. Structural broadband absorbing metamaterial based on three-dimensional printing technology. *Acta Phys. Sin*. **67**, 084202, 10.1007/s12110-009-9068-2 (2018).

Liang, K., Carmone, S., Brambilla, D. & Leroux, J. C. 3D printing of a wearable personalized oral delivery device: A first-in-human study. *Sci Adv* **4**, eaat2544, https://doi.org/ARTN eaat254410.1126/sciadv.aat2544 (2018).

Chai, X. Y. *et al*. Fused Deposition Modeling (FDM) 3D Printed Tablets for Intragastric Floating Delivery of Domperidone. *Sci. Rep*. **7**, 2829, https://doi.org/ARTN 282910.1038/ s41598-017-03097-x (2017).

He, S. *et al*. Low-temperature-cured highly conductive composite of Ag nanowires & polyvinyl alcohol. *Chin. Phys. B* **26**, 078103, https://doi.org/Artn07810310.1088/1674-1056/26/7/078103 (2017).

Liang, Z. *et al*. Facile Synthesis of Nitrogen-Doped Microporous Carbon Spheres for High Performance Symmetric Supercapacitors. *Nanoscale Res. Lett*. **13**, 314–314 (2018).

The correct References are listed below as references 1–5 respectively.
