# Supplementary material for: 3D Printed Polyvinyl Alcohol Tablets with Multiple Release Profiles
Source: Sci Rep. 2019 Aug 28;9:12487. doi: 10.1038/s41598-019-48921-8 (PMC6713737; doi:10.1038/s41598-019-48921-8)
Supplement: Supplementary file 1 — Supplementary Information [file 41598_2019_48921_MOESM1_ESM.doc]

Supplementary information for

**3D Printed Polyvinyl Alcohol Tablets with Multiple Release Profiles**

Xiaowen Xu^1^, Jingzhou Zhao^1^, Maonan Wang^1^, Liang Wang^2^, Junliang Yang^1*^

^1^School of Physics and Electronics, Central South University, Changsha, Hunan, 410083, China

^2^School of Medical Informatics, Xuzhou Medical University, Xuzhou, Jiangsu, 221000, China

*Corresponding author J.L.Y., E-mail address: [junliang.yang@csu.edu.cn](mailto:junliang.yang@csu.edu.cn)


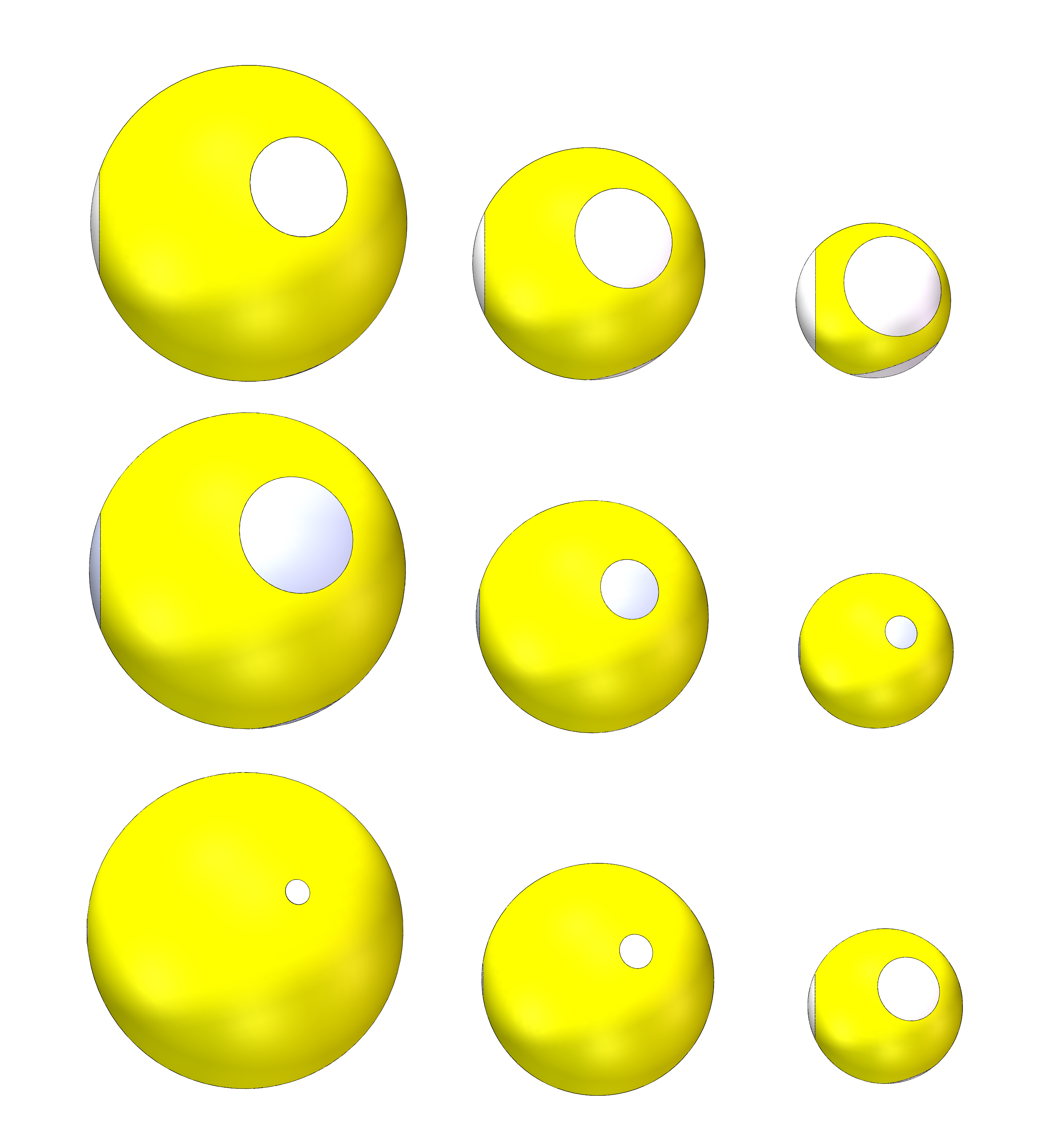


(a)

(b)

(c)

**Fig.S1.** Simulation of drug dissolution process for three kinds of tablets. (a) Cylinder model, (b) Horn model, (c) R-Horn model. The white part is the inner medicine core; the orange part is the outside shell.
